# Effects of Alkyl Ester Chain Length on the Toughness of PolyAcrylate-Based Network Materials

**DOI:** 10.3390/polym15102389

**Published:** 2023-05-20

**Authors:** Yutaro Kawano, Hiroshi Masai, Shintaro Nakagawa, Naoko Yoshie, Jun Terao

**Affiliations:** 1 Department of Basic Science, Graduate School of Arts and Sciences, The University of Tokyo, 3-8-1, Komaba, Meguro-ku, Tokyo 153-8902, Japan; 2 PRESTO, Japan Science and Technology Agency, 4-1-8, Honcho, Kawaguchi, Saitama 332-0012, Japan; 3 Institute of Industrial Science, The University of Tokyo, 4-6-1 Komaba, Meguro-ku, Tokyo 153-8505, Japan

**Keywords:** poly(methyl acrylate), polymer networks, fracture energy, glass transition temperature, polymer toughness, radical polymerization

## Abstract

Polyacrylate-based network materials are widely used in various products owing to their facile synthesis via radical polymerization reactions. In this study, the effects of alkyl ester chains on the toughness of polyacrylate-based network materials were investigated. Polymer networks were fabricated via the radical polymerization of methyl acrylate (MA), ethyl acrylate (EA), and butyl acrylate (BA) in the presence of 1,4-butanediol diacrylate as a crosslinker. Differential scanning calorimetry and rheological measurements revealed that the toughness of MA-based networks drastically increased compared with that of EA- and BA-based networks; the fracture energy of the MA-based network was approximately 10 and 100 times greater than that of EA and BA, respectively. The high fracture energy was attributed to the glass transition temperature of the MA-based network (close to room temperature), resulting in large energy dissipation via viscosity. Our results set a new basis for expanding the applications of polyacrylate-based networks as functional materials.

## 1. Introduction

Polymer network materials are widely used in the industry because of their high toughness and elasticity [1,2,3,4,5,6]. Their broad range of applications is attributed to the diverse chemical and physical properties of their various polymer chains. Poly(alkyl acrylate)s, such as poly(ethyl acrylate)s and poly(butyl acrylate)s, are easily accessible polymers exhibiting high applicability to various polymerization reactions, including free-radical and living-radical polymerizations [7,8,9,10,11,12,13]. However, poly(alkyl acrylate)-based materials exhibit relatively weaker toughness compared with polymethacrylate, polybutadiene, and polystyrene-based materials [14,15,16,17] and have been seldom used as tough materials; Poly(ethyl acrylate)s and poly(butyl acrylate)s are used as adhesives [18,19,20]. Recently, the toughness of polyacrylate-based network materials has been enhanced by block or random copolymerization with other polymer chains and interpenetrations with polymer networks, such as double-network materials [21,22,23,24,25,26,27,28,29,30,31,32,33,34]. Hence, despite the significant potential of poly(alkyl acrylate)-based network materials, simple methodologies for enhancing their toughness are limited. Therefore, the development of a simple and efficient method for increasing the fracture energy of poly(alkyl acrylate)-based polymer networks could expand their use in strong structural and functional materials.

Herein, the systematic investigation of alkyl ester groups in polyacrylate-based network materials revealed a specific toughening effect in poly(methyl acrylate) compared with other alkyl esters. To date, systematic evaluations of the effects of alkyl groups of polyacrylate-based elastic network materials on their toughness have been rarely reported, while the effects of alkyl groups of linear poly(alkyl acrylate)s [35,36,37,38] and rigid networks [39] have been examined. Our investigation revealed that poly(methyl acrylate) exhibited the highest fracture energy among the polyacrylate-based network materials composed of methyl, ethyl, and butyl esters, which were prepared by the radical copolymerization of alkyl acrylates and 1,4-butanediol diacrylate as a crosslinking agent. The enhancement of toughness was attributed to the higher glass transition temperature (near room temperature) of the poly(methyl acrylate) network material compared with the others.

## 2. Materials and Methods

### 2.1. Materials

Methyl acrylate (MA) and 2,2′-azobis(2,4-dimethylvaleronitrile) (ADVN) were purchased from FUJIFILM Wako Pure Chemical. Ethyl acrylate (EA), *n*-butyl acrylate (BA), and 1,4-butanediol diacrylate were purchased from Tokyo Chemical Industry. Dimethylformamide (DMF) was purchased from KANTO CHEMICAL. All reagents and solvents were commercially obtained and used as received.

### 2.2. Preparation of Polymer Networks

The polymer networks were prepared via a general free-radical polymerization process according to a previously published procedure [13]. As shown in Table 1, the reaction solution composed of the monomer (MA, EA, or BA, 11.2 mmol), 1,4-butanediol diacrylate (0.0005, 0.0020, or 0.010 eq.), and ADVN (0.011 mmol) in DMF (200 μL) was degassed in triplicate using the freeze-thaw technique. The solution was injected into a polytetrafluoroethylene (PTFE)-coated reaction mold (40 × 40 × 0.5 mm^3^). The mold was composed of two PTFE-coated glass slides with a 0.5 mm thick spacer, and the glass slides and spacer were held with binder clips (Figure S1). The reaction solution was then placed in an oven (60 °C, 18 h) for polymerization, affording films as network materials containing DMF. After polymerization, the obtained network materials were placed in a vacuum oven at 120 °C for 12 h to remove the remaining solvents and monomers, thus providing elastomeric films.

### 2.3. Characterization

Differential scanning calorimetry (DSC) measurements were performed using Shimadzu DSC-60A Plus under nitrogen flow. Approximately 10 mg of polymer network materials were loaded into aluminum pans. For the measurement of the glass transition temperature, the samples were first heated to 120 °C at 10 °C/min and equilibrated for 20 min to remove thermal history. A subsequent cool/heat cycle was carried out at 5 °C/min. The *T*_g_ data presented in the main text was taken from the second heating curves.

Tensile tests were performed with a Shimadzu EZ-SX tester equipped with a 50 N load-cell. The dumbbell-shaped sample (ISO 37-4 shrunk by 2/3, the initial length of the parallel section was 8 mm) with a thickness of 0.3−0.4 mm was cut from the polymer network materials. The test piece was stretched at a constant crosshead speed at 5 mm/min, 50 mm/min, and 500 mm/min until the test piece failure. At least three test pieces were tested at room temperature, and their mean and standard error were calculated.

The linear viscoelastic properties of the network materials were investigated using a TA Instruments HR30 rheometer equipped with a convection oven and a liquid nitrogen cooling system. The round sample (a radius of 9 mm) with a thickness of 0.3−0.4 mm was cut from the polymer network materials and placed in a parallel plate geometry with a radius of 8 mm. The storage modulus (*G*′) and loss modulus (*G*″) were measured with sinusoidal oscillatory shear at a constant strain amplitude (0.2% or 0.5%) and varying frequency.

The analytical size-exclusion chromatography of linear polymers was performed with a GL-Science GL-7400 HPLC System equipped with Shodex KF-802, -803, -804 columns, a GL-7410 HPLC pump, a GL-7400 UV detector, and a GL-7454 RI detector using THF as the eluent at a flow rate of 0.6 mL/min.

## 3. Results and Discussion

### 3.1. Preparation of Network Materials

**Figure 1:**
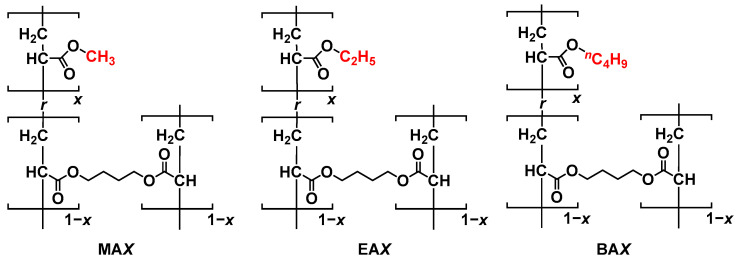
Chemical structures of MA*X*, EA*X*, and BA*X*. (X = 005, 020, and 100 in the case of *x* = 0.0005, 0.0020, and 0.010, respectively).

As shown in Figure 1, three types of poly(alkyl acrylate) network materials were prepared via free-radical polymerization. We polymerized 11 mmol of MA, EA, and BA monomers with 1,4-butanediol diacrylate as a crosslinker in the presence of a radical initiator (ADVN, 0.1 mol%) in DMF at 60 °C for 18 h [40]. Three types of crosslinking densities (0.0005, 0.0020, and 0.010 eq.) were introduced to each poly(alkyl acrylate) network. Consequently, nine types of network materials were prepared with different alkyl chains and crosslinking densities, named MA*X*, EA*X*, and BA*X*, where *X* is 005, 020, and 100 (Table 1). Polymerization proceeded in a polytetrafluoroethylene (PTFE)-coated reaction mold (40 × 40 × 0.5 mm^3^) affording films as network materials containing DMF.

After polymerization, the obtained films were vacuum-dried to remove the residual monomers and solvents because they possibly influence the mechanical properties of the network materials during the heating processes. The vacuum-drying of the films was conducted at 120 °C for 12–18 h, resulting in a 17–19% decrease in material mass. The complete removal of the solvent and monomer was confirmed from the constancy of the material mass. After vacuum drying, transparent elastic films of a 0.3–0.4 mm thickness were obtained as network materials (Figure S2). The films were cut into specimens for characterization.

### 3.2. Thermal Properties of Polymer Materials

The thermal properties of the poly(alkyl acrylate)-based network materials were investigated. The degradation temperatures of MA020, EA020, and BA020 were determined by thermogravimetry (TG) analysis. The 5% weight reduction temperature was greater than 250 °C for all the network materials, indicating their high thermal resistance (Figure S5). Differential scanning calorimetry (DSC) measurements were conducted on nine network samples (MA*X*, EA*X*, and BA*X*; *X* = 005, 020, and 100). The results clearly revealed the reversible base shifts due to glass transitions in all the samples (Figure S4). For example, the glass transition temperature (*T*_g_) of MA020 was at 16.1 °C, which was remarkably higher than that of EA020 and BA020, −14.7 and −50.2 °C, respectively (Table 2). The high *T*_g_ of MA0 was derived from the small mobility of its side chains with methyl groups, which decreased the activation barrier for the segmental motion of the polymer chains [40]. However, the increase of crosslinking density slightly affected the *T*_g_ of the network materials; the *T*_g_ of MA*X*, EA*X*, and BA*X* (*X* = 005, 020, and 100) were in the range of 16 to 18 °C, −15 to −12 °C, and −46 to −50 °C, respectively. These results indicate that the *T*_g_ of the poly(alkyl acrylate)-based network materials is significantly influenced by the type of alkyl ester chains rather than by the crosslinking density. Hence, as the number of carbon atoms on the ester chains decreased, the *T*_g_ of the network materials increased near room temperature, particularly in the case of MA (16–18 °C).

The dependence of *T*_g_ on the alkyl chain length was consistent with the behavior of linear homopolymers without crosslinkers, namely poly(methyl acrylate) (MA0), poly(ethyl acrylate) (EA0), and poly(butyl acrylate) (BA0); the *T*_g_ of the linear polymers increased in the order of BA0 (−54 °C), EA0 (−24 °C), and MA0 (10 °C) as previously reported [40,41]. Notably, the *T*_g_ of the network materials was 3–10 °C higher than that of the respective linear homopolymers, because the presence of crosslinks causes an increase in *T*_g_ [3,42,43]. As a result, the *T*_g_ of MA*X* (*X* = 005, 020, and 100) approached room temperatures because of the crosslinking and the potential high *T*_g_ of poly(methyl acrylate).

### 3.3. Viscoelastic Properties of Polymer Materials

**Figure 2:**
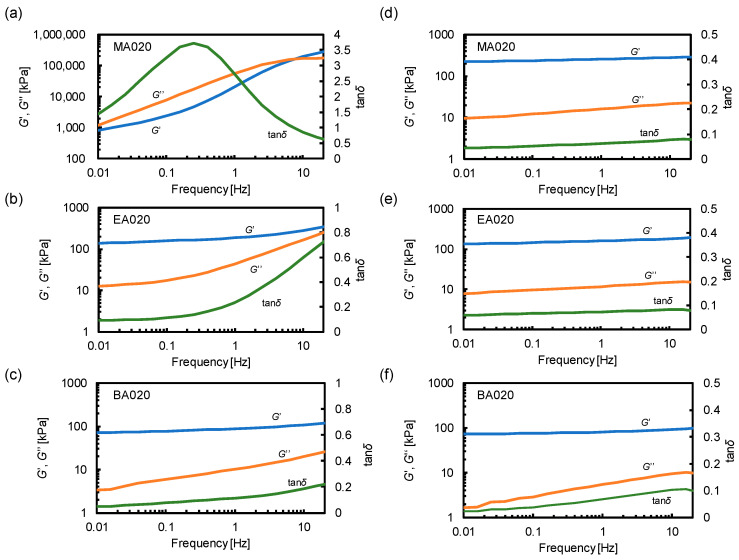
Storage modulus (*G*′), loss modulus (*G*″), and tan*δ* at room temperature for (**a**) MA020, (**b**) EA020, and (**c**) BA020. The *G*′, *G*″, and tan*δ* for (**d**) MA020 measured at 130 °C, (**e**) EA020 measured at 90 °C, and (**f**) BA020 measured at 60 °C.

The dynamic behavior of the poly(alkyl acrylate)-based network materials was investigated by conducting rheological measurements. The frequency-dependent characteristics of MA020, EA020, and BA020 were examined at room temperature as shown in Figure 2a–c. Remarkably, the *G*′ and *G*″ of MA020 at 1 Hz were 100 and 1000 times higher, respectively, than those of EA020 and BA020. Notably, below 10 Hz, only MA020 exhibited a *G*″ larger than *G*′ and the intersection between *G*′ and *G*″ was observed. Consequently, only MA020 exhibited a relaxation as a peak in tan*δ* at 0.3 Hz, indicating segmental motions that resulted in energy dissipation. The viscoelastic differences among MA020, EA020, and BA020 were attributed to the *T*_g_ of MA020 at approximately room temperature.

Additionally, the frequency-dependent moduli of MA020, EA020, and BA020 were measured at temperatures 100–120 °C above the corresponding *T*_g_. This condition enables the removal of the effects of segmental relaxation and highlights the elasticity of the network. *G*′ was independent of the frequency and was much higher than *G*″ for MA020, EA020, and BA020 (Figure 2d–f). This confirms that these materials consist of permanent covalent networks. The rubber elasticity theory states that the shear elastic modulus *G* is determined by the number density of the elastically effective chains *ν*, and is described by Equation (1),

*G* = *νRT*, (1)

where *R* and *T* are the gas constant and temperature, respectively, assuming an affine network model. Equation (1) was used to calculate *ν* from the observed *G* values. The *ν* of MA020, EA020, and BA020 was obtained to be 7.64 × 10^−5^, 5.29 × 10^−5^, and 2.94 × 10^−5^ mol/cm^3^, respectively, showing a decreasing trend upon increase in the length of the alkyl side chain.

To rationalize the difference in the obtained *ν*, we estimated the ideal number density of elastically effective chains (*ν*_ideal_) from the monomer and crosslinker feeds. For considering ideal networks, we assumed that all crosslinkers (1,4-butanediol diacrylate) serve as a 4-branch point and the network is free of any defects such as dangling chains and loops. This led to Equation (2),

*ν_ideal_* = 2*ρx/M*, (2)

where *ρ* is the physical density of the polymer, *x* is the crosslinker-to-monomer ratio, and *M* is the molecular weight of the repeat unit. Detailed calculation procedures can be found in the Supporting Information. The *ν*_ideal_ was determined to be 5.7 × 10^−5^, 4.5 × 10^−5^, and 3.3 × 10^−5^ mol/cm^3^ for MA020, EA020, and BA020, respectively. The order and the trend of the experimental values (*ν*) were generally consistent with those of the ideal values (*ν*_ideal_). From Equation (2), the difference in *ν*_ideal_ among the above three samples comes from the difference in *M/ρ*, which can be recognized as the apparent molar volume per repeat unit. Therefore, the different crosslinking densities and plateau modulus can be explained based on the bulkiness of the repeat units. At the same degree of polymerization, less bulky monomers result in less bulky polymer chains. Therefore, the use of less bulky monomers leads to higher number density of chains and hence higher *ν* in the bulk network material.

### 3.4. Tensile Testing to Reveal the Toughness of the Network Materials

**Figure 3:**
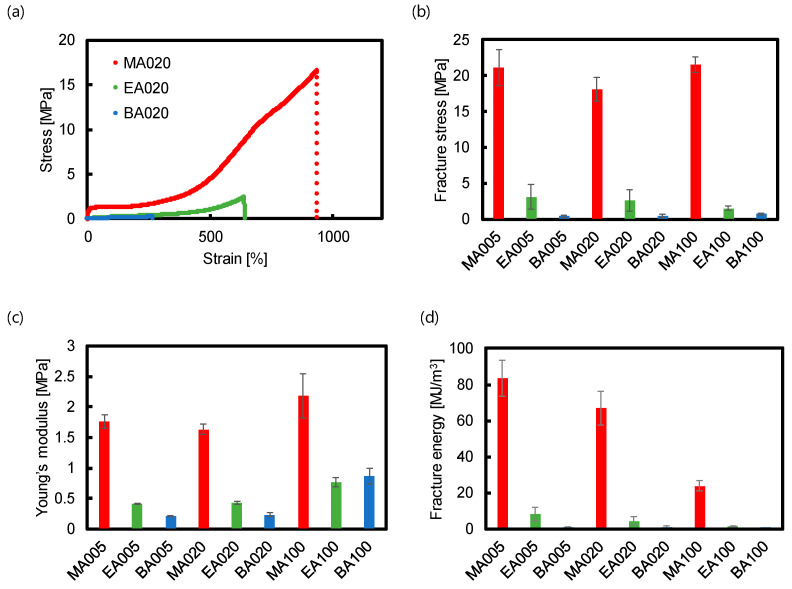
Tensile testing for elastomer materials. (**a**) Representative stress-strain curves for MA020, EA020, and BA020. Comparison of (**b**) fracture stress, (**c**) Young’s modulus, and (**d**) fracture energy between MA*X*, EA*X*, and BA*X*. The tests were conducted in triplicate at room temperature, and their average and standard deviation were calculated.

The mechanical characteristics of the network materials were assessed via uniaxial tensile testing, conducted at an elongation speed of 50 mm/min, which approximately corresponded to the nominal strain rate of 0.10 s^−1^. As shown in Figure 3a and Figure S6, MA*X* exhibited the highest toughness compared with the corresponding EA*X* and BA*X*. The fracture strains of MA*X* (*X* = 020 and 100) were 1.5 times and 2.5 times larger than those of EA*X* and BA*X*, respectively (Figure S7). The fracture strain of MA005 was similar to that of EA005 but 2.5 times larger than that of BA005. In contrast, the fracture stresses of MA*X* significantly surpassed those of EA*X* and BA*X* (Figure 3b). Similarly, MA*X* exhibited a four- and eight-fold higher Young’s modulus compared with EA*X* and BA*X*, respectively, for *X* = 005, 020, and 100 (Figure 3c).

Consequently, as shown in Figure 3d, the fracture energy of MA020 was approximately 10 and 100 times greater than those of EA*X* and BA*X*, respectively, demonstrating the large resistance to mechanical strain. Comparisons of the fracture energies of MA005, MA020, and MA100 revealed that a decrease in the crosslink density increased the fracture energy of the material [44]; MA005 exhibited the highest fracture energy (83 MJ/m^3^). Regardless of the amount of crosslinker, MA*X* exhibited remarkably high fracture energies, which were improved by one or two orders of magnitude compared with the corresponding EA*X* and BA*X*, suggesting the superior mechanical properties of the poly(methyl acrylate)-based network materials.

The superior mechanical performance of MA*X* can be attributed to the unique glass transition temperature of MA-based polymer network materials (close to room temperature); regarding MA020, such a performance was attributed to the methyl ester groups and crosslinking. As mentioned in Section 3.3, a large relaxation via segmental motion was observed in MA*X* at room temperature. The relaxation took place in the time scale ranging from 0.1 to 100 s (corresponding to the frequency of 0.01–10 Hz), which should cover the time scale of the uniaxial tensile tests (~0.1 s^−1^ or ~10 s). Therefore, a considerable amount of energy is absorbed and dissipated by the segmental motion of the poly(methyl acrylate) chains during stretching of the network material, probably owing to the small activation barrier for the segmental motion of the polymer chains derived from the small side-chain methyl groups in MAX [40]. However, EA- and BA-based network materials lack this energy dissipation mechanism due to their *T*_g_, which is much lower than room temperature. The energy dissipation contributes to the high Young’s modulus of the MA-based networks and is also responsible for the large strain at break, leading to the high stress at break and toughness of MA-based networks. In general, the fracture of a polymer network begins with a tiny crack that propagates through the material, eventually leading to catastrophic failure. The extent of the energy concentration at the crack tip is considered to play an important role in the fracture process [45,46,47,48,49]. In MA*X*, the energy dissipation via segmental motions should help dissipate the strain energy at the crack tip, delaying crack propagation and hence the fracture of the material. Consequently, MA*X* exhibited significantly superior strain at break, stress at break, and fracture toughness compared with EA*X* and BA*X*.

To verify the toughening mechanism, the strain rate dependence of the tensile behavior was investigated. MA020, EA020, and BA020 were tested at the additional elongation speed of 5 and 500 mm/min, which approximately corresponded to the nominal strain rate of 0.01 and 1.0 s^−1^, respectively. Figure S8 compares the fracture energies at different elongation speeds derived from the stress-strain curves. The mechanical properties of MA020 and BA020 were independent of the elongation speed, whereas increasing the elongation speed improved the toughness of EA020; the fracture energy of EA020 at 50 mm/min was approximately 3 times higher compared with that at 5 mm/min. The enhancement of the toughness of EA020 can be attributed to the segmental relaxation. In Figure 2b, EA020 showed an upturn in tan*δ* at the high-frequency limit (>1 Hz), indicating that the relaxation via segmental motion becomes more prominent at a shorter time scale. Thus, more energy dissipation takes place at a higher elongation speed, leading to an increased toughness. BA020 did not show an increase in tan*δ* within the frequency range tested (Figure 2c). There was almost no energy dissipation by the segmental motion in BA020 in the accessible time scale, resulting in the independence of the mechanical properties on the elongation speed. In MA020, dynamic moduli and tan*δ* considerably varied with the frequency (Figure 2a). Therefore, it was rather surprising that MA020 did not show the elongation speed dependence. We speculate that the time scale of the tensile tests corresponded to a relatively high-frequency region in the rheological spectra. In Figure 2a, the storage and loss moduli almost become independent of the frequency at >1 Hz. Thus, MA020 had a similar degree of energy dissipation at all elongation speeds, leading to the toughness independent of the elongation speed. These results support the proposed mechanism, i.e., the toughness of poly(alkyl acrylate)-based network materials was drastically enhanced through the energy dissipation from the segmental relaxation.

## 4. Conclusions

In this study, the effects of the alkyl groups of polyacrylate-based network materials on their thermal and rheological properties were systematically evaluated. We synthesized nine types of polymer network materials with different crosslinking densities and different alkyl ester lengths on the polymer side chains: methyl, ethyl, and *n*-butyl esters. The MA network materials exhibited elevated glass transition temperature (*T*_g_), around room temperature, which was attributed to the potentially high *T*_g_ of poly(methyl acrylate) and the crosslinking of the network. Rheological measurements indicated that the loss modulus of the MA-based network materials exceeds their storage modulus at room temperature. Consequently, the materials exhibited high toughness owing to their large fracture energy, which was induced by energy dissipation from the viscosity, compared with other poly(alkyl acrylate)-based counterparts, poly(ethyl acrylate), and poly(butyl acrylate).

## Figures and Tables

**Table 1:** Composition of the reaction solution for the synthesis of the network materials MA*X*, EA*X*, and BA*X*.

Network Material	Monomer (MA, EA, or BA)		Crosslinker (1,4-Butanediol Diacrylate)		Initiator (ADVN)		Solvent (DMF)
	[mL]	[mmol]	[μL]	[mmol]	[mg]	[mmol]	[mL]
MA005	1.00	11	1.05	0.0055	2.8	0.011	0.20
MA020	1.00	11	4.21	0.022	2.8	0.011	0.20
MA100	1.00	11	21.0	0.11	2.8	0.011	0.20
EA005	1.19	11	1.05	0.0055	2.8	0.011	0.20
EA020	1.19	11	4.21	0.022	2.8	0.011	0.20
EA100	1.19	11	21.0	0.11	2.8	0.011	0.20
BA005	1.59	11	1.05	0.0055	2.8	0.011	0.20
BA020	1.59	11	4.21	0.022	2.8	0.011	0.20
BA100	1.59	11	21.0	0.11	2.8	0.011	0.20

**Table 2:** Glass transition temperatures of the network materials MA*X*, EA*X*, and BA*X*.

*X*	*T*_g_ of Network Materials [°C]		
	MA*X*	EA*X*	BA*X*
0 (homopolymer)	10.0^41^	−24.0^41^	−54.0^41^
005	16.5	−15.1	−49.0
020	16.1	−14.7	−50.2
100	17.6	−12.1	−46.0

## Data Availability

Data will be made available on request.

## Supplementary Materials

The following supporting information can be downloaded at: https://www.mdpi.com/article/10.3390/polym15102389/s1, Figure S1. Schematic representation of reaction mold for the synthesis of network materials: Figure S2. The photograph of network materials (MA020, EA020, and BA020) cut out in a round shape: Figure S3. SEC analyses of the polymerization of methyl acrylate, ethyl acrylate, and butyl acrylate without a crosslinker (1,4-butanediol diacrylate) (Detector: RI): Detailed discussion for calculation of ideal number density of elastically effective chains (*ν*_ideal_): Figure S4. DSC thermograms of MA020 during the 2nd and 3rd heating processes: Figure S5. Thermogravimetric analyses of MA020, EA020, and BA020: Figure S6. Representative stress-strain curves for (a) MA005, EA005, and BA005 and (b) MA100, EA100, and BA100: Figure S7. The comparison of fracture strain for MA*X*, EA*X*, and BA*X* (*X* = 005, 020, and 100): Figure S8. Fracture energies of MA020, EA020, and BA020, that were measured with tensile tests at the elongation speed of 5, 50, and 500 mm/min: Figure S9–S23. Stress-strain curves of MA005, EA005, BA005, MA020, EA020, BA020, MA100, EA100, and BA100 [50].
