# Supplementary material for: Effects of Alkyl Ester Chain Length on the Toughness of PolyAcrylate-Based Network Materials
Source: Polymers (Basel). 2023 May 20;15(10):2389. doi: 10.3390/polym15102389 (PMC10224553; doi:10.3390/polym15102389)
Supplement: Supplementary file 1 [file polymers-15-02389-s001.zip › polymers-2391048-supplementary.pdf]

Supplementary Materials

Effects of Alkyl Ester Chain Length on the Toughness of Poly-Acrylate-Based Network Materials

Yutaro Kawano, Hiroshi Masai, Shintaro Nakagawa, Naoko Yoshie and Jun Terao**

Table of Contents

1. Preparation of materials ········· 2
2. Calculation of ideal number density of elastically effective chains (ν_{ideal}) ········· 3
3. Thermal properties of polymer materials ········· 4
4. Tensile testing of the network materials ········· 5
5. Reference ········· 12

1. Preparation of materials

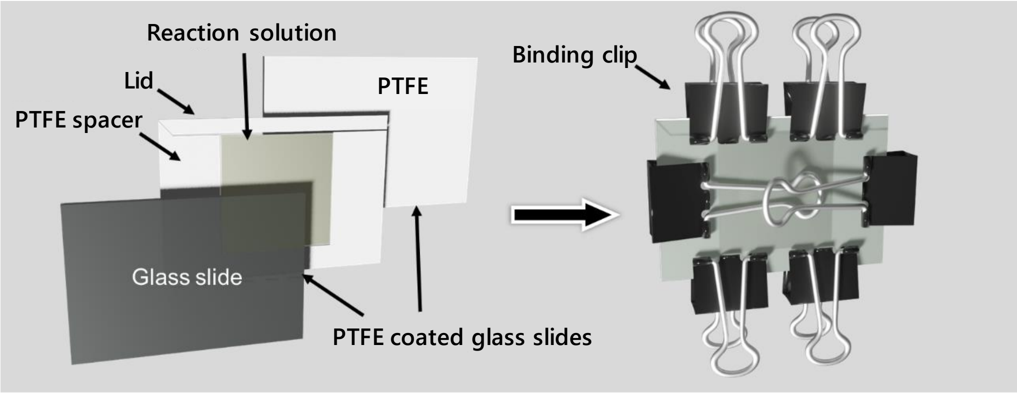

Figure S1. Schematic representation of reaction mold for the synthesis of network materials.

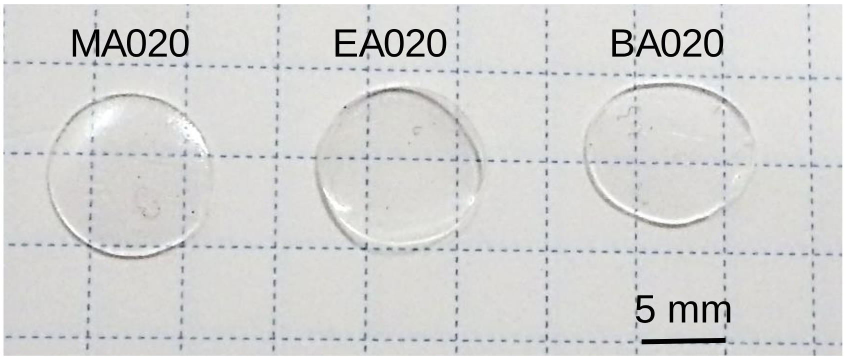

Figure S2. The photograph of network materials (MA020, EA020, and BA020) cut out in a round shape (Scale bar, 5 mm).

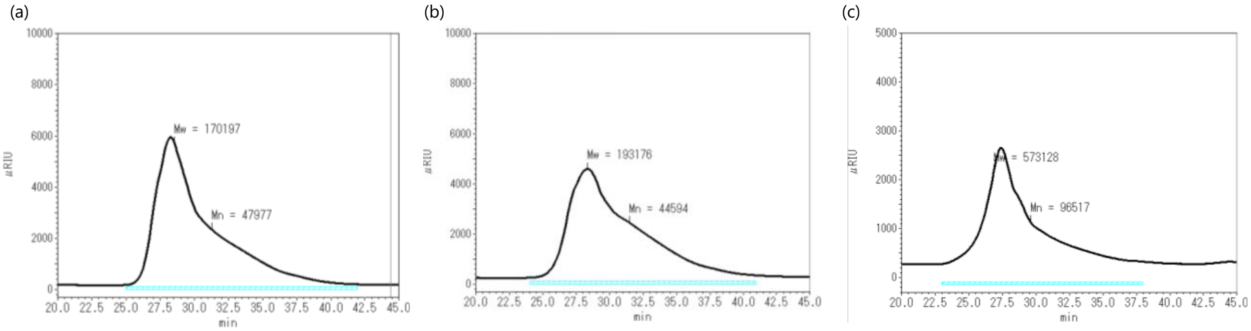

Figure S3. SEC analyses of the polymerization of (**a**) methyl acrylate, (**b**) ethyl acrylate, and (**c**) butyl acrylate without crosslinker (1,4-butanediol diacrylate) (Detector: RI). Except for the absence of a crosslinker, the polymerization condition was the same as for the corresponding network materials.

2. Calculation of ideal number density of elastically effective chains (ν_{ideal})

To explain the effects of the side group bulkiness on ν in MA020, EA020, and BA020, we have estimated the ideal values of ν for each network material based on the aforementioned rubber elasticity theory. We made the following assumptions:

1) All crosslinkers (1,4-butanediol diacrylate) were incorporated into the network and served as 4-branching points.

2) All monomers were consumed in the polymerization.

3) All chains connecting two adjacent crosslinks behave as elastically effective chains. That is, there are no defects such as loops and dangling chains.

4) The volume of the crosslink points is negligible compared to that of the network chains (which is reasonable considering that the amount of the crosslinkers was less than 1% of the monomers).

In this case, the ideal values of ν were estimated as eq (2),

$$\nu_{ideal} = \frac{2\rho x}{M} \quad (2)$$

where ρ, M, and x are the physical density of the polymer (Poly(methyl acrylate), Poly(ethyl acrylate), or Poly(butyl acrylate)), the molecular weight of the repeat unit, and the molar ratio of the crosslinker against the monomer. The density of the corresponding linear polymers (Poly(methyl acrylate): 1.22 g/cm^3, Poly(ethyl acrylate): 1.13 g/cm^3, Poly(butyl acrylate): 1.06 g/cm^3) was used as ρ [1]. Using x = 0.0020, ν_{ideal} of MA020, EA020, and BA020 was calculated as 5.7 × 10^{-5}, 4.5 × 10^{-5}, and 3.3 × 10^{-5} mol/cm^3, respectively, which are in the same order and the trend as the calculated ν from plateau modulus (MA: 7.64 ×10^{-5} mol/cm^3, EA: 5.29 ×10^{-5} mol/cm^3, BA: 2.94 ×10^{-5} mol/cm^3). Hence, the ca. 2-fold difference of crosslinking densities and plateau modulus could be sufficiently explained by the bulkiness of polymer chains. From Equation (2), the difference in ν_{ideal} among the above three samples comes from the difference in M/ρ, which can be recognized as the apparent molar volume per repeat unit. Therefore, the different crosslinking densities and plateau modulus can be explained by the bulkiness of the repeat units; less bulky MA chains increased the polymer density and number density of effective chains (ν).

3. Thermal properties of polymer materials

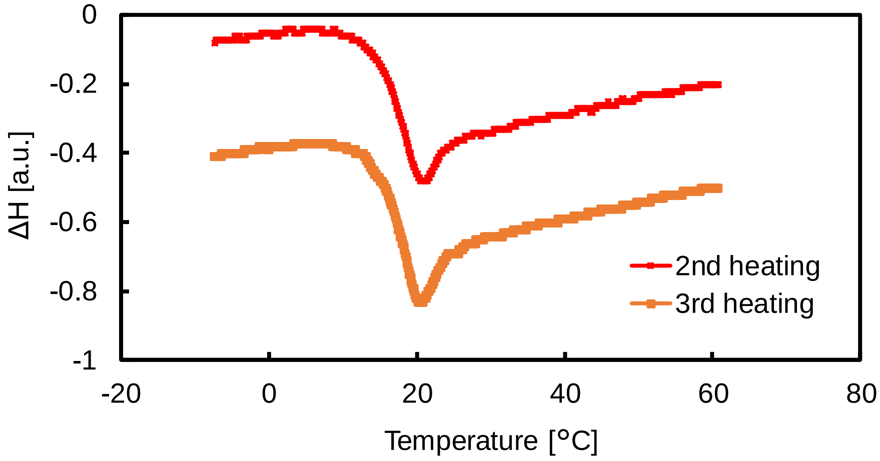

Figure S4. DSC thermograms of MA020 during the 2nd and 3rd heating processes.

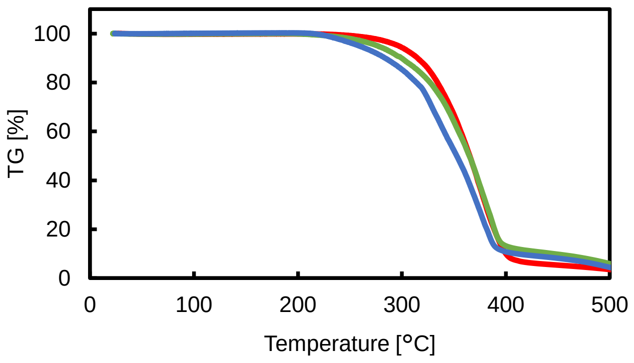

Figure S5. Thermogravimetric analyses of MA020 (red), EA020 (green), and BA020 (blue).

4. Tensile testing of the network materials

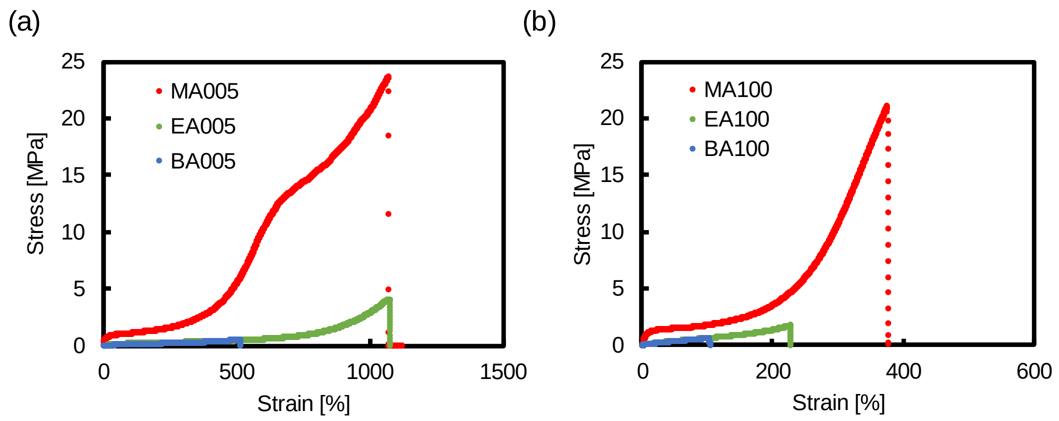

Figure S6. Representative stress-strain curves for (**a**) MA005, EA005, and BA005 and (**b**) MA100, EA100, and BA100.

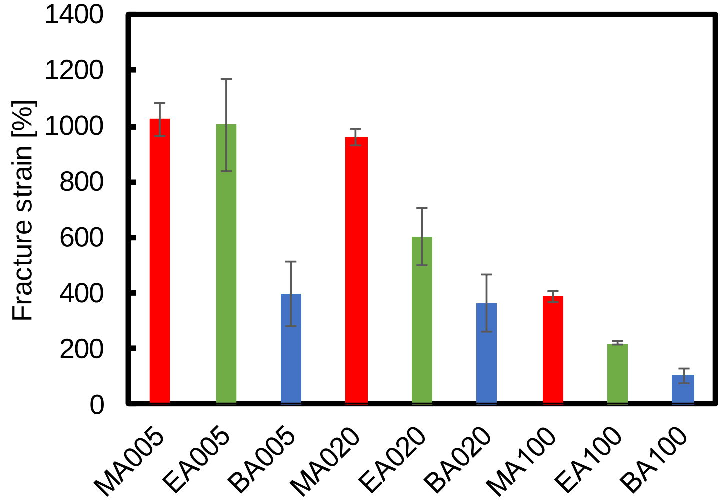

Figure S7. The comparison of fracture strain for MA*X*, EA*X*, and BA*X* (*X* = 005, 020, and 100).

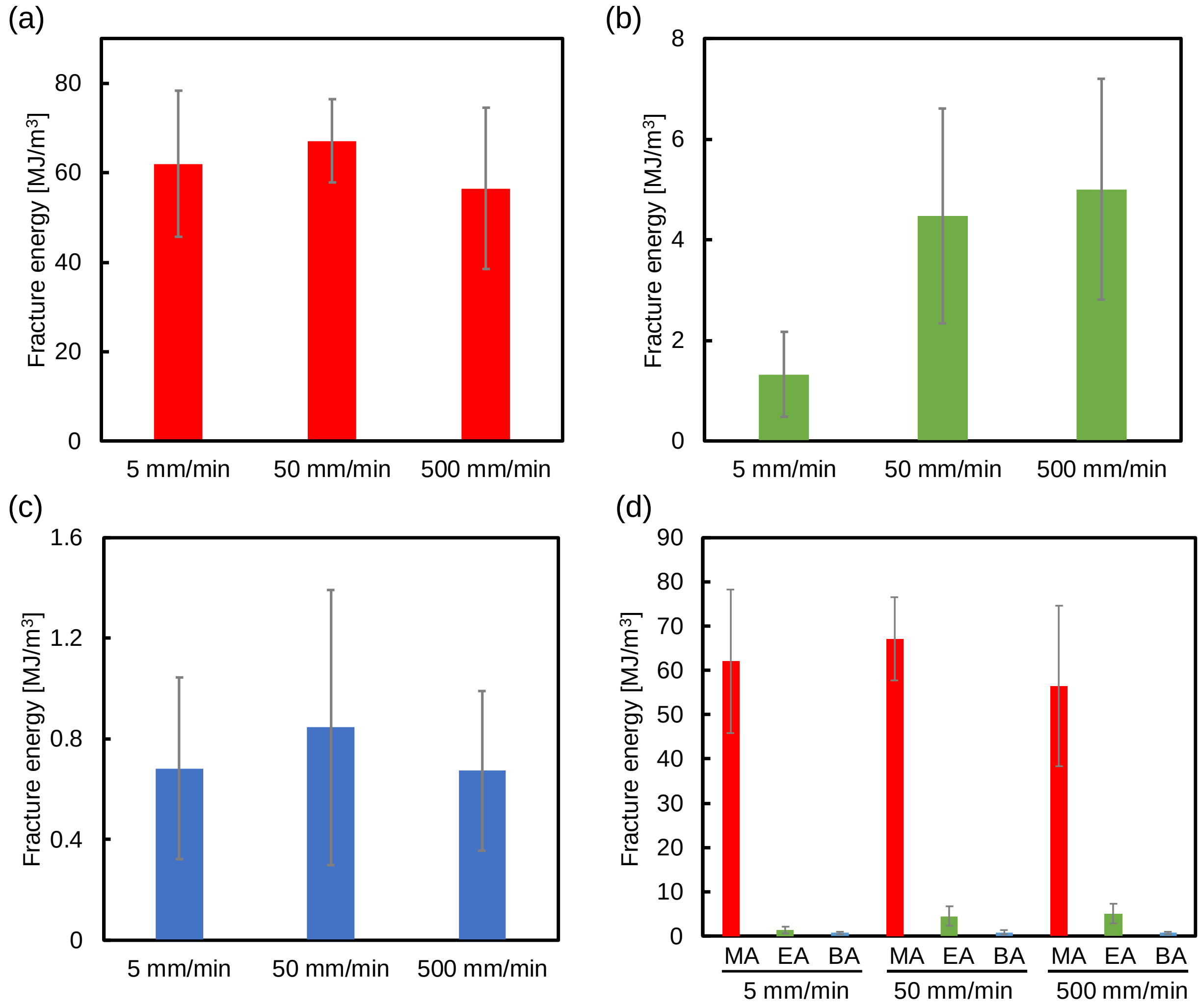

Figure S8. Fracture energies of (**a**) MA020, (**b**) EA020, and (**c**) BA020 measured with tensile tests at the elongation speed of 5, 50, and 500 mm/min. (**d**) Summary of the elongation speed-dependence of fracture energies in (**a**–**c**).

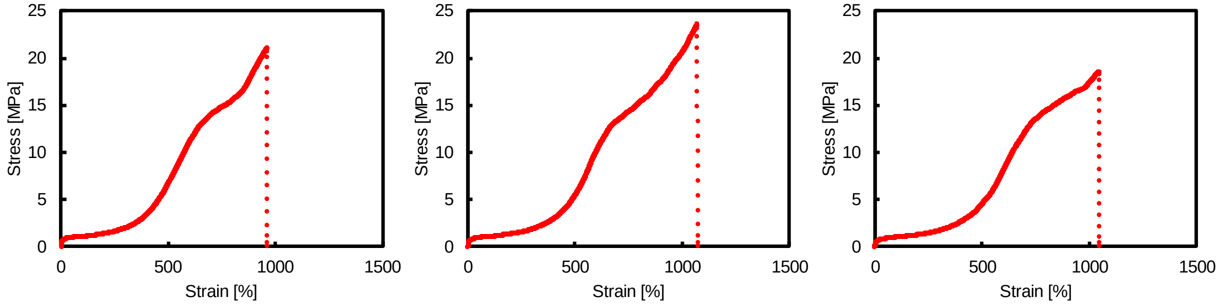

Figure S9. Stress-strain curves of three samples of MA005 at the elongation speed of 50 mm/min.

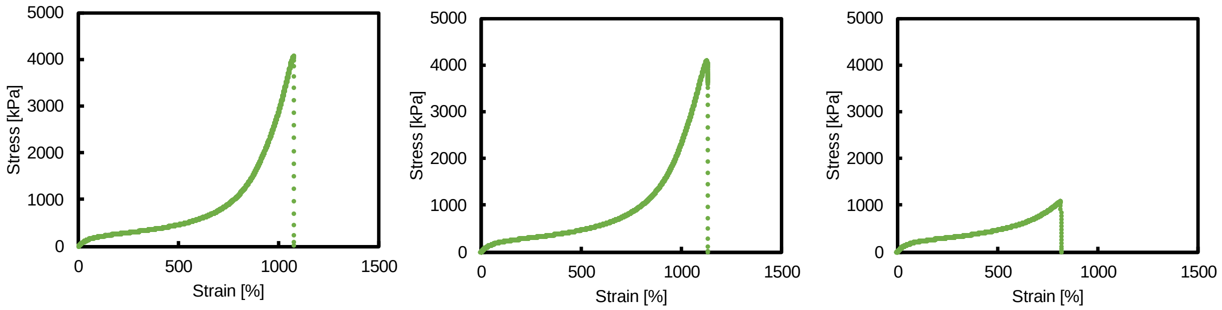

Figure S10. Stress-strain curves of three samples of EA005 at the elongation speed of 50 mm/min.

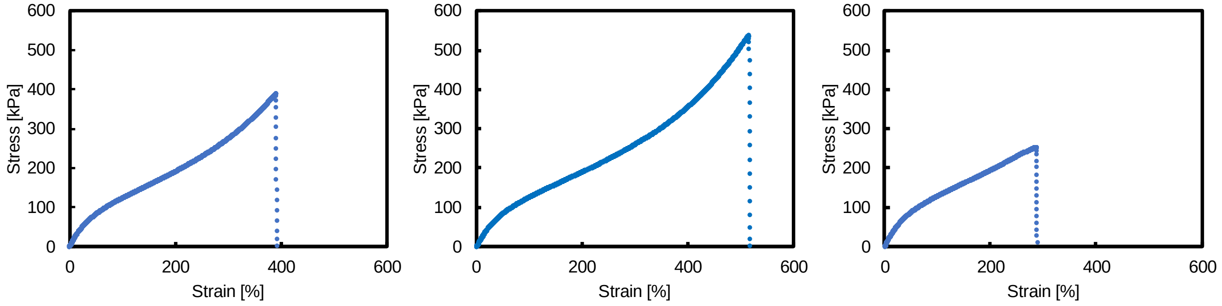

Figure S11. Stress-strain curves of three samples of BA005 at the elongation speed of 50 mm/min.

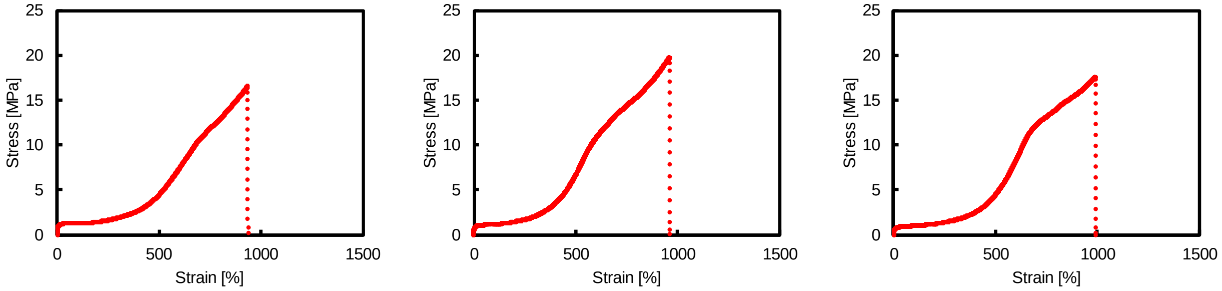

Figure S12. Stress-strain curves three samples of MA020 at the elongation speed of 50 mm/min.

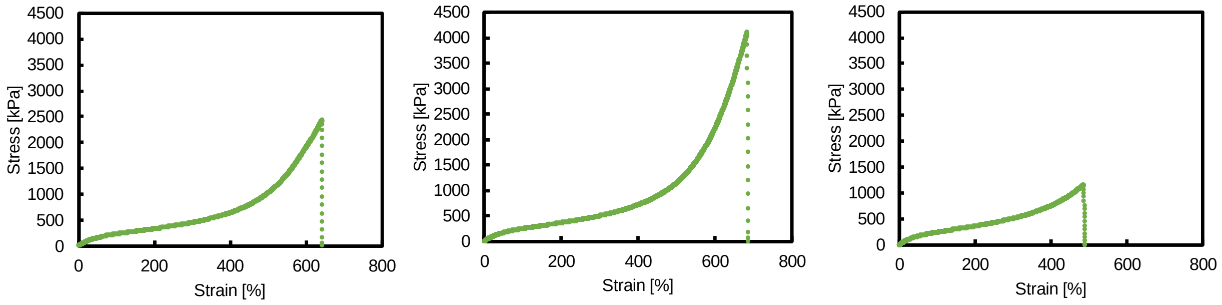

Figure S13. Stress-strain curves of three samples of EA020 at the elongation speed of 50 mm/min.

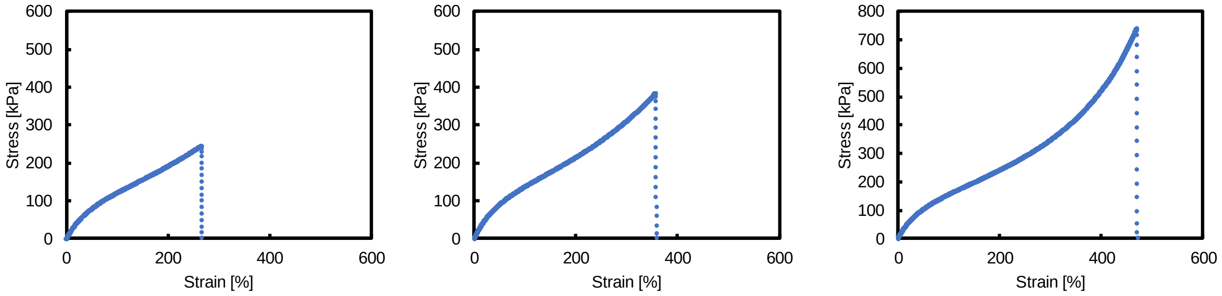

Figure S14. Stress-strain curves of three samples of BA020 at the elongation speed of 50 mm/min.

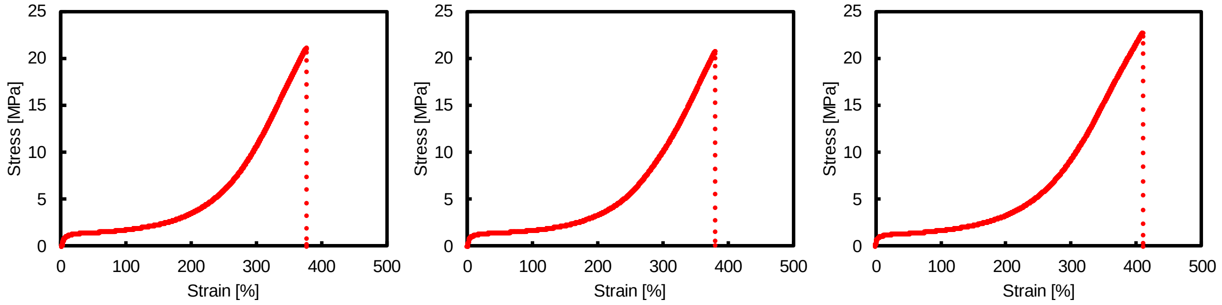

Figure S15. Stress-strain curves of three samples of MA100 at the elongation speed of 50 mm/min.

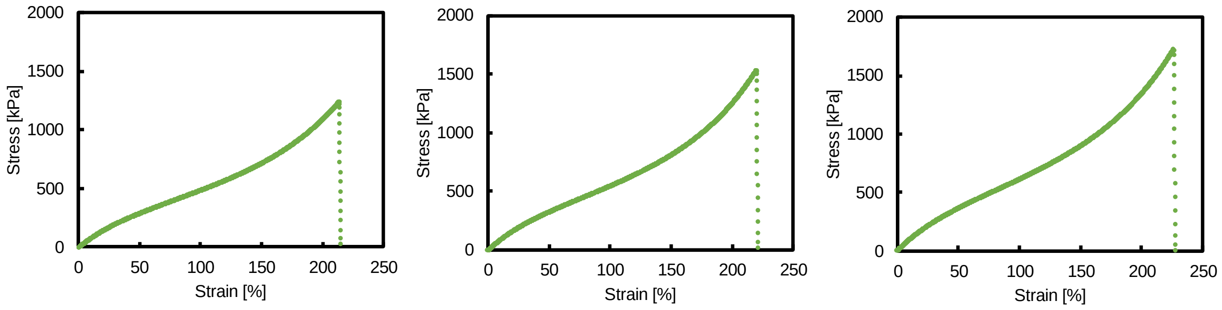

Figure S16. Stress-strain curves of three samples of EA100 at the elongation speed of 50 mm/min.

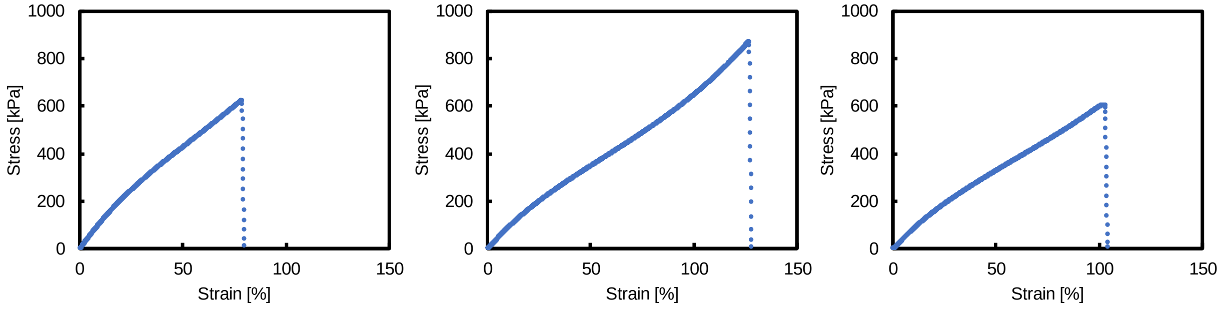

Figure S17. Stress-strain curves of three samples of BA100 at the elongation speed of 50 mm/min.

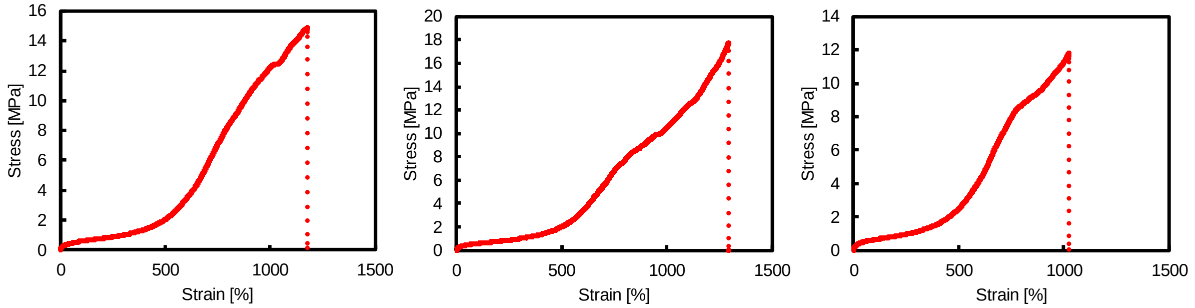

Figure S18. Stress-strain curves three samples of MA020 at the elongation speed of 5 mm/min.

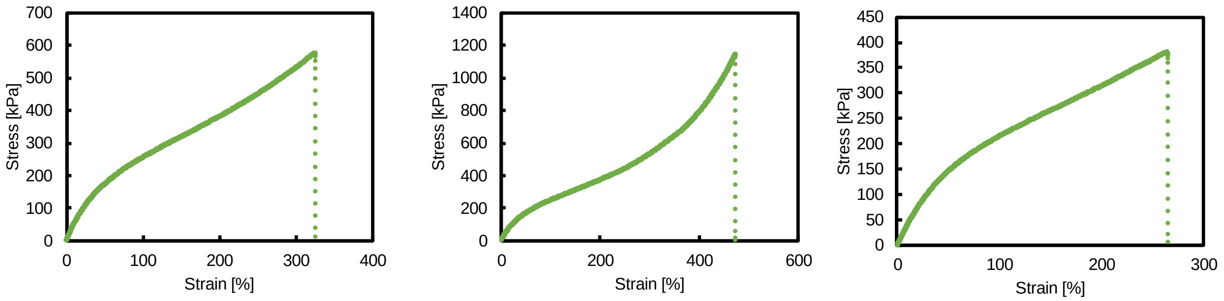

Figure S19. Stress-strain curves of three samples of EA020 at the elongation speed of 5 mm/min.

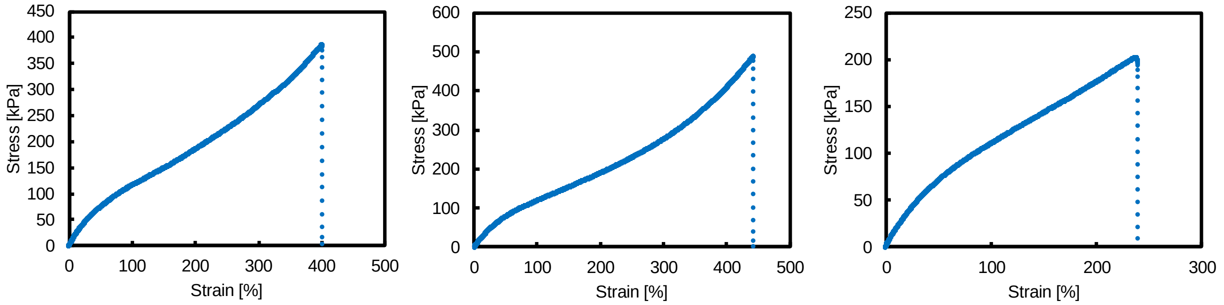

Figure S20. Stress-strain curves of three samples of BA020 at the elongation speed of 5 mm/min.

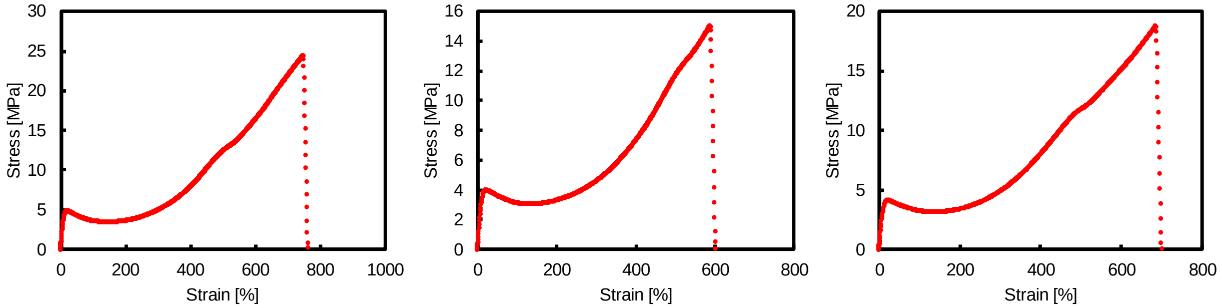

Figure S21. Stress-strain curves three samples of MA020 at the elongation speed of 500 mm/min.

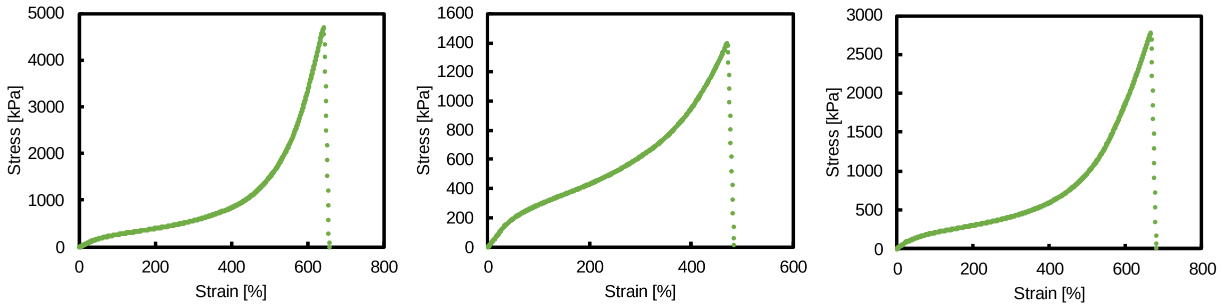

Figure S22. Stress-strain curves three samples of EA020 at the elongation speed of 500 mm/min.

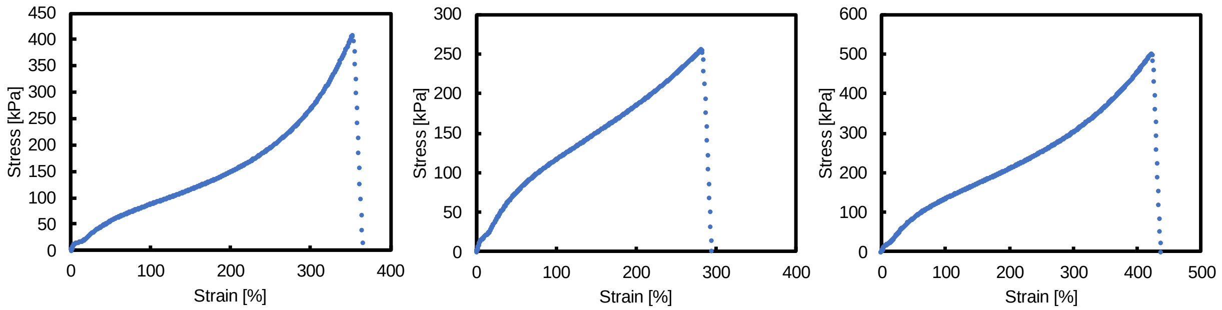

Figure S23. Stress-strain curves three samples of BA020 at the elongation speed of 500 mm/min.

5. Reference

[1] Mashita, K.; Hirooka, M. Alternating copolymers of isobutylene and acrylic ester by complexed copolymerization. *Polymer*, **1995**, *36*, 2983–2988.
